# Lithium-Cation Conductivity of Solid Solutions in Li_6-x_Zr_2-x_A_x_O_7_ (A = Nb, Ta) Systems

**DOI:** 10.3390/ma14226904

**Published:** 2021-11-16

**Authors:** Georgiy Sh. Shekhtman, Anastasia V. Kalashnova, Boris D. Antonov

**Affiliations:** Institute of High Temperature Electrochemistry, Ural Branch, Russian Academy of Sciences, 20 Akademicheskaya St., 620990 Ekaterinburg, Russia; kalashnova@ihte.uran.ru (A.V.K.); B.Antonov@ihte.uran.ru (B.D.A.)

**Keywords:** solid electrolytes, lithium zirconates, lithium cation conductivity, crystal structure, glycine-nitrate synthesis, heterovalent doping

## Abstract

Li_6-x_Zr_2-x_AxO_7_ (A = Nb; Ta) system with 0 < x < 0.30 is synthesized by glycine-nitrate method. Boundaries of solid solutions based on monoclinic Li_6_Zr_2_O_7_ are determined; temperature (200–600 °C) and concentration dependences of conductivity are investigated. It is shown that monoclinic Li_6_Zr_2_O_7_ exhibits better transport properties compared to its triclinic modification. Li_5.8_Zr_1.8_Nb(Ta)_0.2_O_7_ solid solutions have a higher lithium-cation conductivity at 300 °C compared to solid electrolytes based on other lithium zirconates due the “open” structure of monoclinic Li_6_Zr_2_O_7_ and a high solubility of the doping cations.

## 1. Introduction

Lithium zirconates are a subject of wide-ranging studies during the last years due to the wide range of their practical applications. In particular, their feasibility of using as tritium breeder materials in thermonuclear fusion reactors [1,2,3], and carbon dioxide sorbents [4,5], has been demonstrated. Besides, lithium zirconates are thermodynamically stable against Li [6], so may be used in lithium power sources. So, attempts have been made to use lithium zirconates as component of electrode mass [7,8,9,10,11] and solid lithium conducting electrolytes [12,13] for various types of lithium batteries. For effective application of lithium zirconates data on their conductivity is of great importance. However, data on lithium zirconates conduction and influence of doping are rather scarce and literature data are contradictory.

According to [14,15], in Li_2_O–ZrO_2_ system form following compounds: Li_2_ZrO_3_, Li_6_Zr_2_O_7_, and Li_8_ZrO_6_. Initial study of Li_2_ZrO_3_ and Li_8_ZrO_6_ conductivity revealed that their Li^+^-ion conductivity is 10^−3^–10^−4^ S·cm^−1^ at 400 °C and due to their high activation energy they show quick lowering with drop of temperature [12,16]. In more recent publications it was reported that Li_2_ZrO_3_ near 430 °C transforms into superionic state with sharp rise in ionic conductivity and a heavy drop in activation energy from 98.4 to 13.8 kJ·mol^−1^ [17]. A jump-like growth in Li ion conductivity at 400–470 °C was also recorded for Li_8_ZrO_6_ [18]. For that reason, the authors supposed that both compounds transform into superionic state with disordering of Li^+^ sublattice. However, efforts to stabilize high-temperature phases at room temperature by doping, were not successful [19,20]. Besides that, the heavy rise in Li_2_ZrO_3_ conductivity at 430 °C was not reproduced later [13].

Li^+^ ion conductivity of Li_6_Zr_2_O_7_ was first measured in [12]. The authors aimed to synthesize Li_4_ZrO_4_, but later investigations show that lithium orthozirconate does not exist, and received X-ray patterns earlier ascribed to Li_4_ZrO_4_ [21] belong to Li_6_Zr_2_O_7_ [15]. The conductivity of Li_6_Zr_2_O_7_ measured in [12] is rather low: 3.0 × 10^−6^ S·cm^−1^ at 300 °C. However, subsequently it was found that the conductivity of Li_6_Zr_2_O_7_ can be considerably increased by heterovalent doping. Thus, when lithium is substituted for divalent magnesium or zinc cations, the conductivity of the resulting solid electrolytes is 10^−4^ at 300 °C [22]. A more considerable conductivity growth is observed when Zr^4+^ ions are substituted for pentavalent cations (Nb^5+^ or Ta^5+^) with the positive charge excess compensation by lithium vacancies. Lithium-cation conductivity of Li_5.85_Zr_1.85_Ta_0.15_O_7_ solid solution is above 10^−3^ S·cm^−1^ at 300 °C [23], (Table 1).

On substitution of Li^+^ by double charged ions and on substitution of Zr^4+^ by pentavalent ions Nb^5+^ or Ta^5+^, charge compensation is accomplished by the creation of lithium vacancies. Electromigration in that kind of solid solutions is realized via the vacancy mechanism, i.e., lithium vacancies are the charge carriers. The amount of vacancies increases with the concentration of the dopant and depends on the range of the single phase region. However, the work [23], investigates only two compositions of solid electrolytes: Li_5.85_Zr_1.85_A_0.15_O_7_ (A = Nb, Ta). Such high conductivity values obtained in the initial studies give reason to extend study of the conductivity of Li_6_Zr_2_O_7_-based solid solutions in Li_6-x_Zr_2-x_Nb_x_O_7_ and Li_6-x_Zr_2-x_Ta_x_O_7_ systems in the wide concentration region.

The present paper investigates the effect the concentration of pentavalent doping cations in Li_6-x_Zr_2-x_A_x_O_7_ (A = Nb, Ta) systems has on the lithium-cation conductivity of Li_6_Zr_2_O_7_. It is assumed that when A^5+^ ions replace Zr^4+^, the compensation of the charge imbalance by lithium vacancies can be described as

A_2_O_5_ + 2Li_2_O → 2A_Zr_^•^+ 4Li_Li_^×^ + 7O_o_^×^ + 2V_Li_^′^. → Li_6_Zr_2_O_7_ (1)

## 2. Materials and Methods

Li_2_CO_3_ (reagent grade), Zr(OH)_2_CO_3_·5.5H_2_O (analytical grade), and Nb_2_O_5_ or Ta_2_O_5_ (analytical grade) all VECTON RF were used as starting reagents to synthesize the substances under study. Lithium carbonate was previously dried at 300 °C, niobium and tantalum oxides were annealed at 1000 °C.

Several methods of Li_6_Zr_2_O_7_ synthesis are described in literature: solid state reaction [15,24,25,26], co-precipitation of hydroxides [5], a combined method [27]. In [24], solid solutions based on Li_6_Zr_2_O_7_ were produced via urea combustion followed by heat treatment. A similar method is used in the present work, except for the fact that instead of urea we use glycine.

The required quantities of the starting reagents were weighed (FX40-CJ analytical balance, Tokyo, Japan BMI Surplus) within the accuracy of ±10^−4^ g and ground together in a jasper mortar. To compensate for Li_2_O loss at heat treatment, a slight (5 wt%) excess of Li_2_CO_3_ was added to the starting mixtures. The resulting mixtures were dissolved in a water solution of HNO_3_ (reagent grade), evaporated under stirring, then glycine (C_2_H_5_NO_2_) of analytical purity was added. After portion of water evaporates, the reaction mixtures turn into a syrup-like liquid, which ignites spontaneously at further heating. The powder obtained was calcinated in air at 650 °C for 3 h to remove the organic compounds, then the mixtures were ground and sintered at 850–950 °C for 8–16 h. The obtained materials were ground into powder with particles less than 50 µm and pressed in stainless steel die into pellets 10 mm in diameter and 1–1.5 mm thick at 100–300 MPa. The pressed pellets were sintered at 900 °C for 5 h in the powder of the same composition. The density of the sintered pellets was 90–95% of the theoretical.

The phase composition of the samples was studied by XRD on a Rigaku D/MAX-2200VL/PC instrument (RIGAKU, Tokyo, Japan) in filtered Cu Kα-radiation generated at 40 kW, 30 mA (*λ* = 1.54178 Å) stepwise with 0.3 s counting time and the step of 0.02° at room temperature in air. MDI Jade 6.5 software and PDF-2 ICDD database were used to analyze phase composition and to calculate unit cell parameters. The error of the cell parameters calculation did not exceed 0.02%. The data for full-profile Rietveld analysis in the range of 15° < 2Θ < 90° were recorded with 4 s holding time per step. Crystal structure refinement was performed using the FullProf program [28].

The morphology and microstructure of the material synthesized were examined using MIRA 3 LMU scanning electron microscope (TESCAN, Brno, Czech Republic).

**Figure 1:**
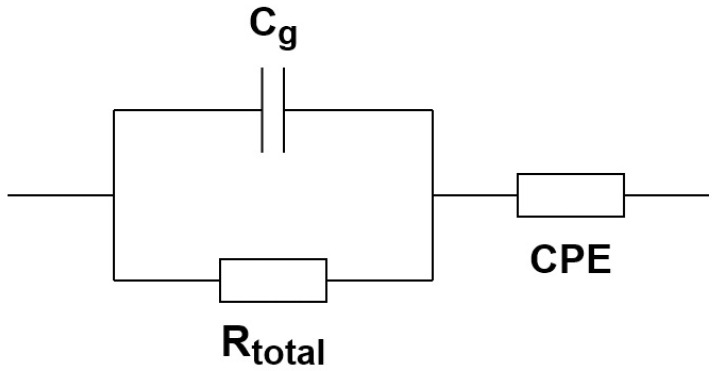
Resultant equivalent circuit for Ag│Li_6-x_Zr_2-x_Nb(Ta)_x_O_7_│Ag cell.

The electrical resistance of the samples was measured in air across 200–600 °C temperature range by P-40xpotentiostat-galvanostat (Elins, Zelenograd, Russia) with FRA-24M module for electrochemical impedance measurements over the frequency range of 50 Hz–500 kHz. Silver applied by a thermochemical method was used as electrodes. The measurements were performed during stepwise cooling with the step of 20 °C. The samples were kept for 15–20 min at every measurement temperature to allow the electric resistance to reach a stable value. The resistance of the samples was determined by analyzing the impedance frequency dispersion. The resultant equivalent circuit is given in Figure 1, where C_g_ is the geometrical capacity, R_total_ is the resistance of the sample, CPE is the constant phase element reflecting the polarization at the electrode–electrolyte interface. The resistance of the sample in this case is the sum of the volume resistance (R_v_) and grain boundary resistance (R_gb_), i.e., R_total_ = R_v_ + R_gb_.

**Figure 2:**
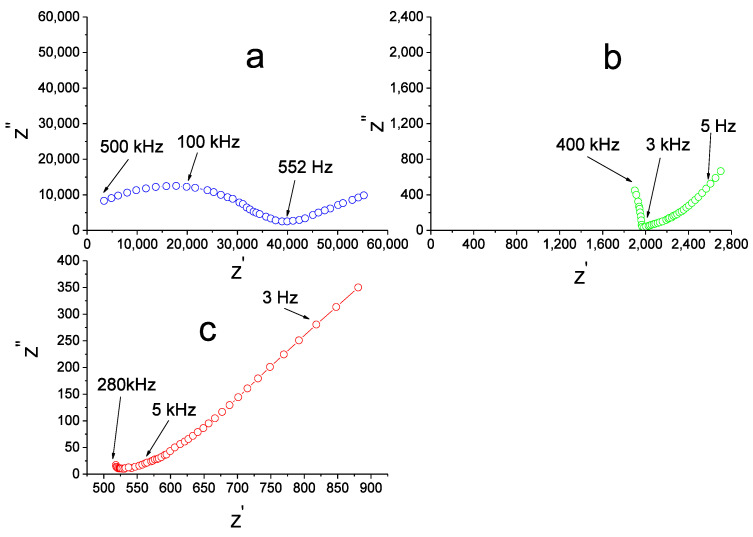
Impedance spectra of Ag|Li_5.95_Zr_1.95_Nb_0.05_O_7_| Ag cell at 225 (**a**), 400 (**b**), 550 (**c**).

The impedance spectra for the undoped Li_6_Zr_2_O_7_ and for the solid solutions are identical. The spectra for the Ag|Li_5.95_Zr_1.95_Nb_0.05_O_7_|Ag cell at different temperatures are given in Figure 2 as examples. At low temperatures the spectra consist of a semicircular arc in the high-frequency region and a tail in the low-frequency region (Figure 2a).

The resistance R_total_ of the sample was determined as the value corresponding to the point of intersection between the high-frequency arc and the Z^’^ axis. As the temperature rises, the semicircle becomes smaller (Figure 2b), and above 500–550 °C only a tail is observed across the whole accessible frequency range (Figure 2c). In this case R_total_ was found by the extrapolation of the low-frequency linear portion onto the Z^’^ axis. The corresponding value of specific conductivity was calculated according to the formula:

σ = l/R_total_·S (2)

where l is the length of the sample, S is the area of its cross-section.

The electron conductivity was determined using DC method with gold electrodes at 40–50 mV and in all the cases did not exceed 1% of total conductivity.

## 3. Results and Discussion

The crystal structure of Li_6_Zr_2_O_7_ was studied by X-ray analysis of single crystals [27,29], in [26], the X-ray method was enlarged with the neutron diffraction in order to refine the positions of Li^+^ ions. These data show a good agreement. Li_6_Zr_2_O_7_ has a monoclinic structure, space group C2/c, a = 10.4428(1) Ǻ, b = 5.9877(1) Ǻ, c = 10.2014(1) Ǻ, β = 100.266(1)° [25]. The ions of oxygen form a distorted cubic close-packed lattice, in which 1/8 of the sites are vacant. The distribution of lithium and zirconium ions is similar to the distribution of cations in NaCl; besides, zirconium is octahedrally coordinated by oxygen, and Li coordination number is five (a square pyramid).

According to [21], at low temperatures there exists a metastable α-form of Li_6_Zr_2_O_7_, which is produced by annealing the mixture of Li_2_CO_3_ and ZrO_2_ at 850–900 °C and which passes into β-form at high temperatures. The work [15] confirms the existence of α-modification of Li_6_Zr_2_O_7_, which has a triclinic structure with a = 6.0153(4) Å, b = 9.1941(12) Å, c = 5.3112(6) Å, α = 96.30(1)°, β = 107.16(1)°, γ = 89.74(1)°, however, the authors point out that this phase can only be formed if Li_2_CO_3_ is used as lithium source. Synthesis at 750 °C produces single-phase Li_6_Zr_2_O_7_ samples with triclinic structure. If the annealing temperature is 800 °C, the triclinic and monoclinic modifications co-exist in the sample, and at higher temperatures the triclinic one disappears. Using ZrO_2_ and Li_2_O oxides as the starting components yielded a monoclinic phase irrespective of the synthesis temperature. Thus, the formation of the metastable triclinic modification of Li_6_Zr_2_O_7_ via synthesis from Li_2_CO_3_ and ZrO_2_ is confirmed by literature data, but the temperature and the time of heat treatment required to produce single-phase samples with a stable monoclinic structure are quite different in [15,22].

**Figure 3:**
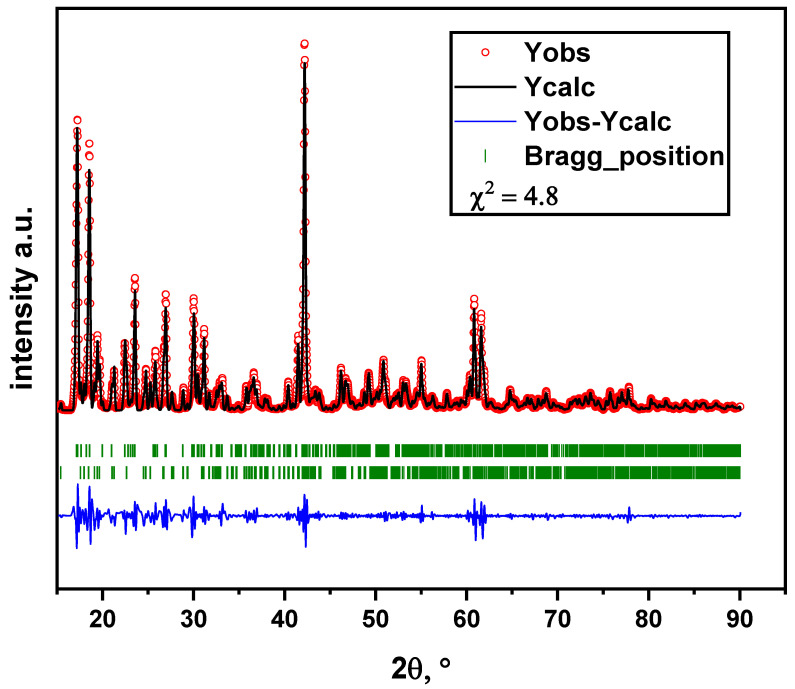
Rietveld refinement of powder XRD pattern for Li_6_Zr_2_O_7_ sintered at 900 °C for 8 h. Dots represent the observed data, solid line marks the calculated data. Short green ticks indicate the peak positions of Bragg reflections; the upper row of short green ticks marks the monoclinic phase; the lower row of short green ticks marks the triclinic phase. The bottom line indicates the difference between the calculated and observed data.

In the present work, the glycine-nitrate stage of Li_6_Zr_2_O_7_ synthesis was followed by keeping the reaction mixture at 850, 900, and 950 °C. The results indicate that heat treatment at 850 and 900 °C produces a mixture of triclinic and monoclinic modifications. Notably, if the heat treatment time is increased from 8 to 16 h, the proportion of the triclinic modification decreases. Figure 3 contains Rietveld refinement of the powder XRD pattern for the sample sintered at 900 °C for 8 h. The data indicate that Li_6_Zr_2_O_7_ powder is non-single-phase. The main phase is characterized by a monoclinic syngony with space group C2/c (PDF No 88-2213). The impurity phase of the same chemical composition has a triclinic syngony with space group P1 (PDF No 36-0122). The content of the triclinic phase is estimated to be 8.9%.

**Figure 4:**
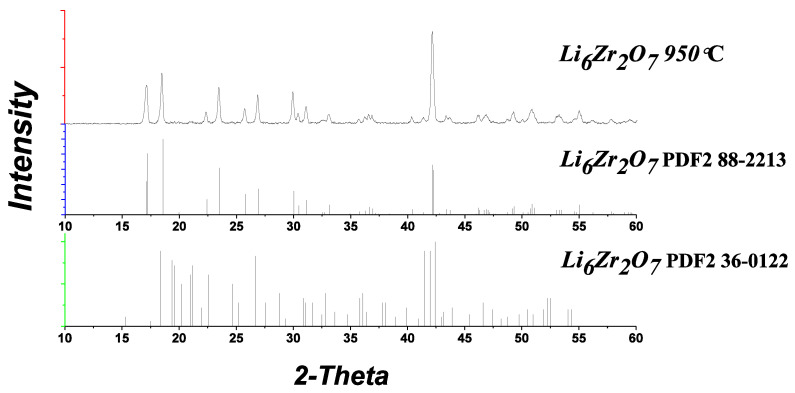
XRD patterns for Li_6_Zr_2_O_7_ obtained by glycine-nitrate method from Li_2_CO_3_ and Zr(OH)_2_CO_3_·5.5H_2_Oat 950 °C.

The XRD patterns of the samples annealed at 950 °C contain only the reflections of monoclinic Li_6_Zr_2_O_7_ (Figure 4), the same applies to the doped samples.

**Figure 5:**
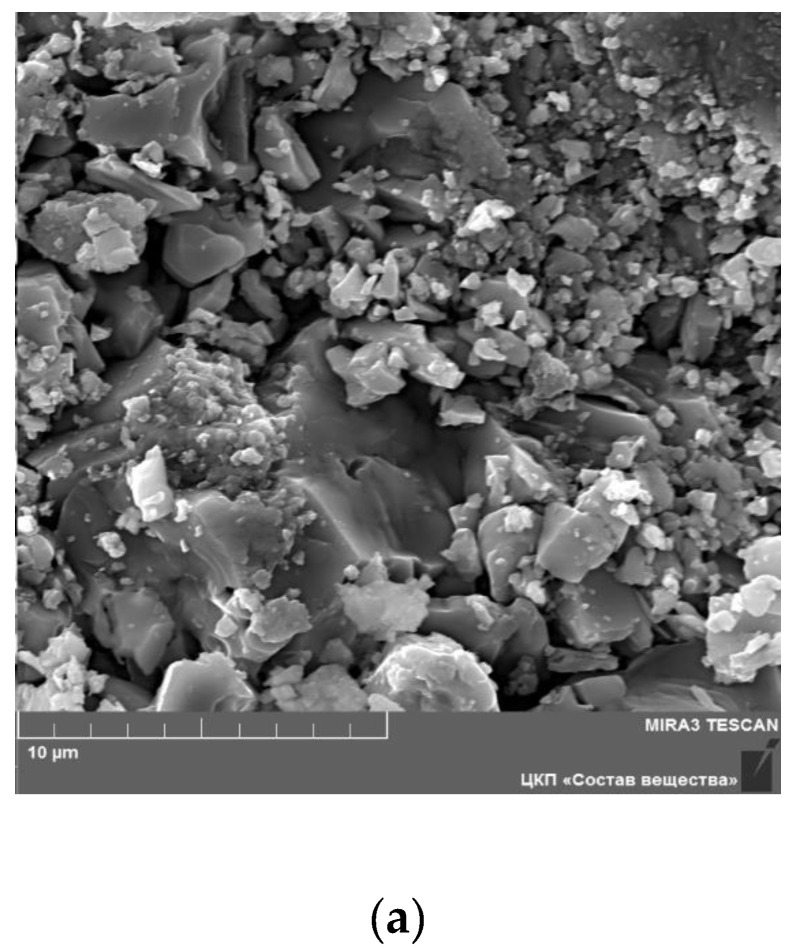

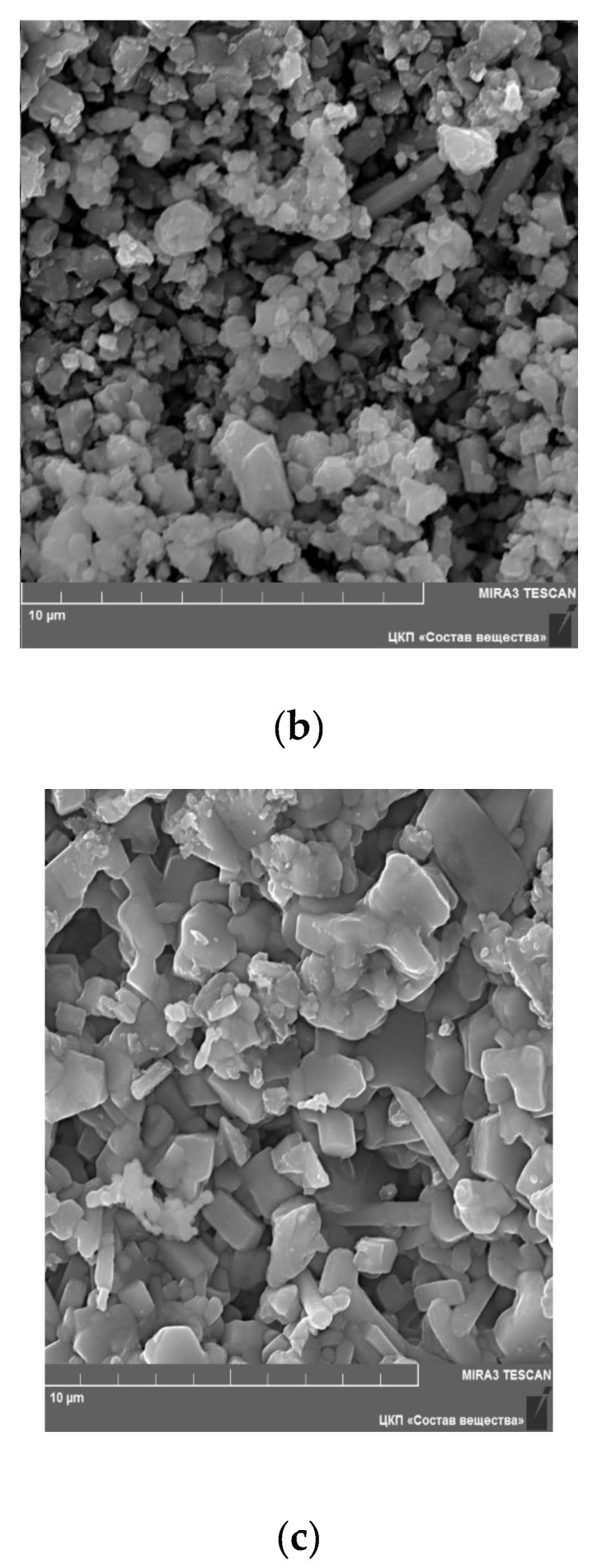
SEM images for the surface of Li_6_Zr_2_O_7_ (sintered at 950 °C) (**a**), Li_5.8_Zr_1.8_Ta_0.2_O_7_ (**b**), and Li_5.8_Zr_1.8_Nb_0.2_O_7_ sample (**c**).

Figure 5 shows SEM images for the surface of Li_6_Zr_2_O_7_ sample annealed at 950 °C, as well as for Li_5.8_Zr_1.8_Ta_0.2_O_7_ and Li_5.8_Zr_1.8_Nb_0.2_O_7_ solid solutions. One can see that the microstructure of the samples is non-homogeneous, and in the case of Li_6_Zr_2_O_7_ it consists mainly of particles 0.5–2.0 µm in size (Figure 5a), though some grains are as large as 3 µm. In Li_5.8_Zr_1.8_Ta_0.2_O_7_ and Li_5.8_Zr_1.8_Nb_0.2_O_7_ the structure is more homogeneous and consists of grains 1–2 µm in size (Figure 5b,c).

It should be pointed out that at temperatures of 900 °C and above lithium oxide is quite volatile, therefore, increasing the temperature and time of heat treatment may cause a considerable loss of Li_2_O. On the other hand, decreasing the temperature results in the formation of triclinic phase in the samples. Thus, a question arises about how the presence of the triclinic modification of Li_6_Zr_2_O_7_ affects the conductivity, i.e., what is the relation between the conductivity of the samples having triclinic and monoclinic structure.

**Figure 6:**
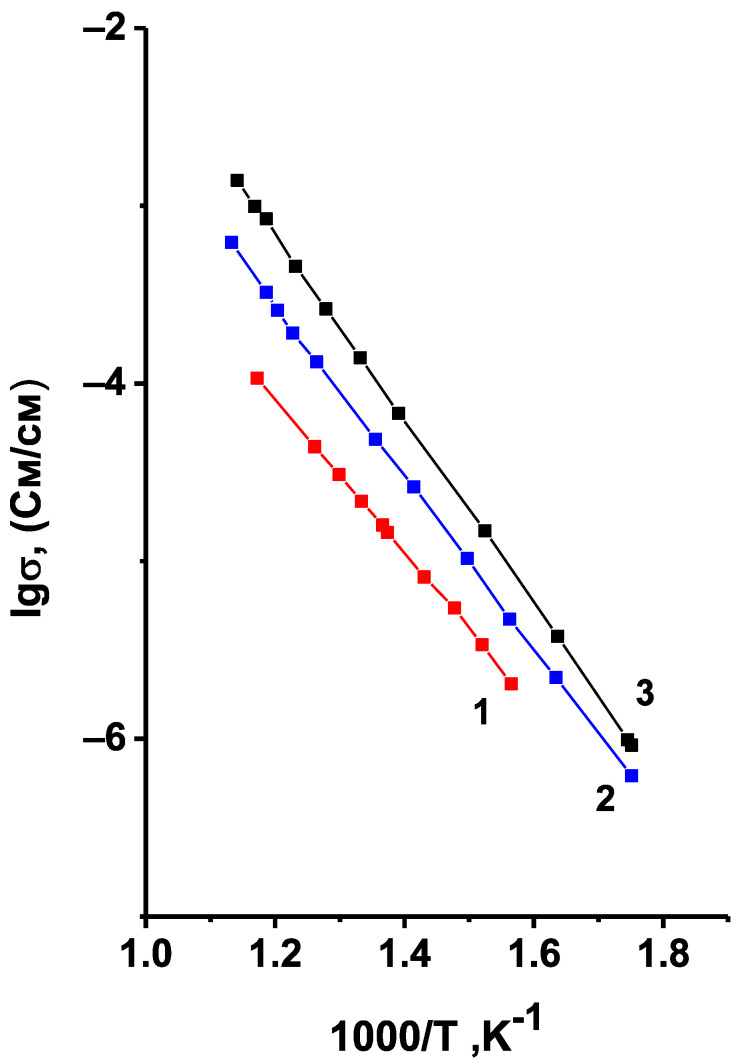
Temperature dependences of conductivity for Li_6_Zr_2_O_7_ samples synthesized at 850 °C (1), 900 °C (2), 950 °C (3).

Figure 6 shows the temperature dependences of conductivity for Li_6_Zr_2_O_7_ samples synthesized at different temperatures. Lines 1 and 2 correspond to the two-phase samples that contain triclinic phase alongside the monoclinic one. It can be assumed from the intensity of the reflections on the XRD pattern, that the content of triclinic phase in sample 1, synthesized at 850 °C, is higher than in sample 2, synthesized at 900 °C. Line 3 corresponds to the single-phase sample with monoclinic structure. Its electric characteristics are close to those given in [12] (Table 1).

**Figure 7:**
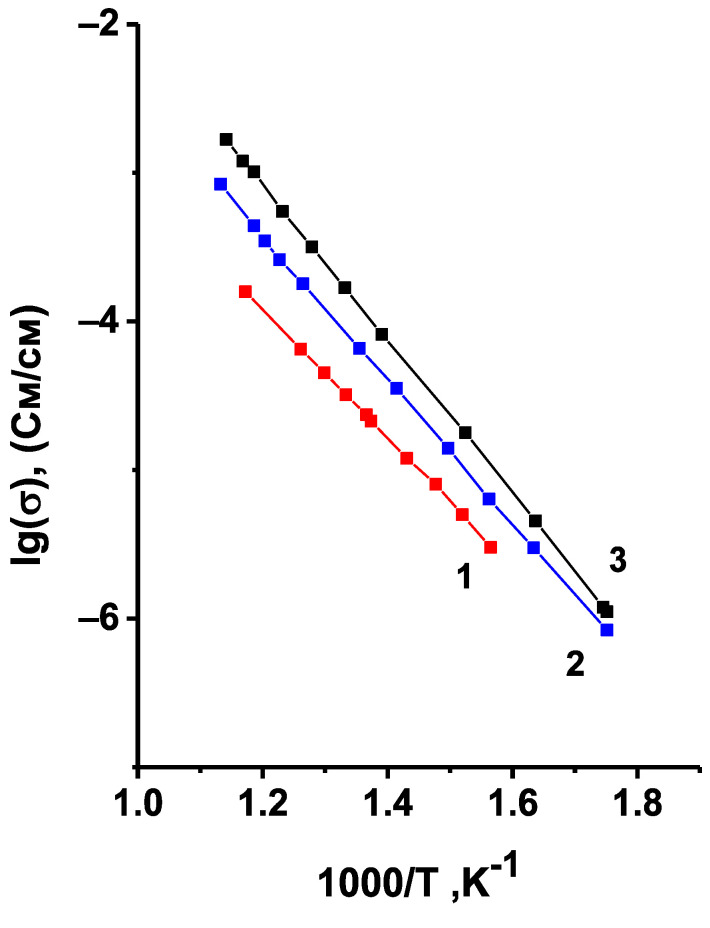
Temperature dependences of Li_6_Zr_2_O_7_ conductivity corrected to zero porosity of ceramic samples. The temperature of synthesis and annealing is 850 (1), 900 (2), 950 °C (3).

As can be seen from Figure 6, the conductivity of Li_6_Zr_2_O_7_ decreases with the appearance of triclinic phase and with the further growth of its content. However, one should take into account that the samples sintered at lower temperatures have a higher porosity, e.g., the porosity of the samples sintered at 850, 900, and 950 °C assessed from saturating the samples with an organic liquid was 24, 19, and 13%, respectively. An increase in the porosity of the samples always leads to a decrease in their conductivity, while the triclinic modification of Li_6_Zr_2_O_7_ may have both higher and lower conduction as compared to the monoclinic one. To get reliable data on the relation between the conductivity of triclinic and monoclinic forms of Li_6_Zr_2_O_7_, it is necessary to eliminate the effect of porosity. In order to do that we corrected the calculation of conductivity for samples 1, 2, and 3 to the conductivity of non-porous ceramics using the formula for heterogeneous materials [30]:

σ*=σ12σ1+σ2σ2−σ1+2m2−1.65π(σ2−σ1)4σ1+3σ2m21032σ1+σ2σ2−σ1−m2−1.65π3(σ2−σ1)4σ1+3σ2m2103

where σ^*^ is the measured value of conductivity, *σ*_1_ is the conductivity of the non-porous sample, *m*_2_ is the volume fraction of pores, *σ_2_* in this case is the conductivity of air, i.e., *σ*_2_ = 0. The temperature dependences of the corrected conductivity values are given in Figure 7. One can observe here the same tendency for the conductivity to decrease with decreasing synthesis temperature, and, consequently, with the increasing content of the triclinic modification. Thus, across the investigated temperature range the triclinic modification of Li_6_Zr_2_O_7_ has a much lower conductivity compared to the monoclinic one, therefore, the final temperature of synthesis for the doped samples was 950 °C, and the absence of the triclinic phase in the synthesized samples was monitored by XRD.

**Figure 8:**
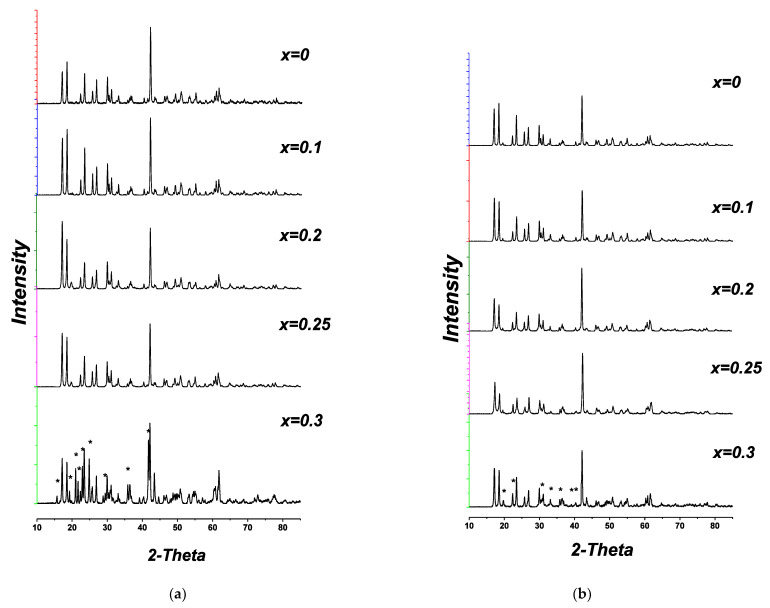
XRD patterns for the samples in Li_6-x_Zr_2-x_A_x_O_7_ system, A = Nb (**a**), Ta (**b**).

Phase diagrams for Li_2_O-ZrO_2_-Nb_2_O_5_ (Ta_2_O_5_) systems were investigated in [31], but the authors focused mainly on the study of perovskite-like phases near LiNbO_3_ and LiTaO_3_, and, as a result, the domain of Li_6-x_Zr_2-x_Nb(Ta)_x_O_7_ was not considered. According to our results, in both systems under investigation, i.e., Li_6-x_Zr_2-x_A_x_O_7_ (A = Nb, Ta), the samples with 0 <x< 0.25 retain the monoclinic structure of the initial Li_6_Zr_2_O_7_ (Figure 8a,b, XRD patterns for x = 0–0.20).

When x ≥ 0.25, reflections of impurity phases appear on the XRD patterns of the samples, their intensity grows with increasing content of the dopants (Figure 8a,b, XRD patterns for x = 0.25, 0.30). In Li_6-x_Zr_2-x_Nb_x_O_7_ system these reflections correspond to the recently investigated Li_29_Zr_9_Nb_3_O_40_ compound [32], (PDF 02-082-2342). The phase appearing in the samples of Li_6-x_Zr_2-x_Ta_x_O_7_ with x ≥ 0.25 could not be identified using PDF 2, but all the lines that cannot be assigned to Li_6_Zr_2_O_7_ practically coincide with the reflections of Li_29_Zr_9_Nb_3_O_40_. Taking into account the closeness of chemical properties that have niobium and tantalum and close values of ionic radii for Nb^5+^ and Ta^5+^, we can assume that there exists a tantalum analog isostructural with Li_29_Zr_9_Nb_3_O_40_. The results of XRD analysis for the samples of the systems studied are listed in Table 2.

**Figure 9:**
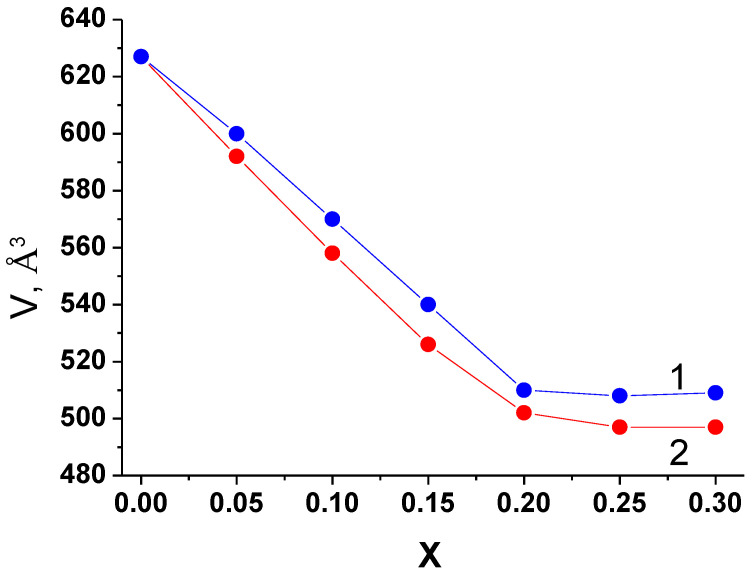
Concentration dependence of the volume of elementary cells in the monoclinic solid solutions of Li_6-x_Zr_2-x_Nb_x_O_7_ (1) and Li_6-x_Zr_2-x_Ta_x_O_7_ (2).

The plots of concentration vs. the volumes of elementary cells for the phases with monoclinic C2/c structure in the systems investigated are given in Figure 9. The radii of Nb^5+^ and Ta^5+^ ions are practically the same (0.78 Å), but they are slightly smaller than Zr^4+^ (0.86 Å) [33], therefore, the volumes of the elementary cells decrease with the growth of A^5+^ content, and for the samples with the same value of *x* in the systems with niobium and tantalum their values are close. Overall, the plots of *V* vs. *x* correlate with the results of the phase composition studies (Table 2).

**Figure 10:**
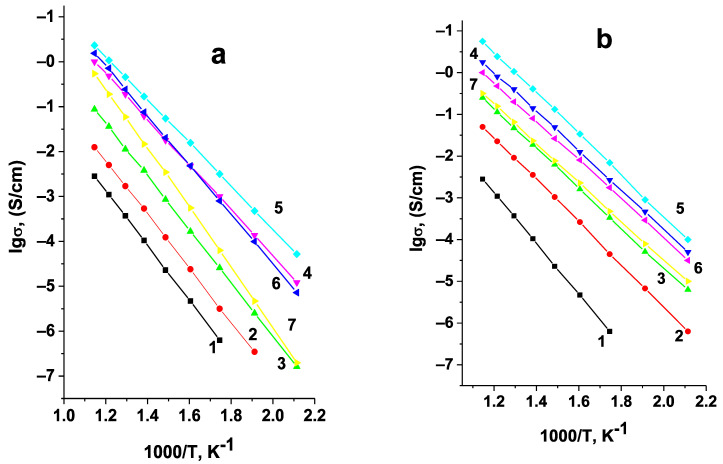
Arrhenius plots for conductivity of Li_6-x_Zr_2-x_A_x_O_7_ A = Nb (**a**), Ta (**b**); x = 0(1); 0.05(2); 0.10(3); 0.15(4); 0.20(5); 0.25(6); 0.30(7).

**Figure 11:**
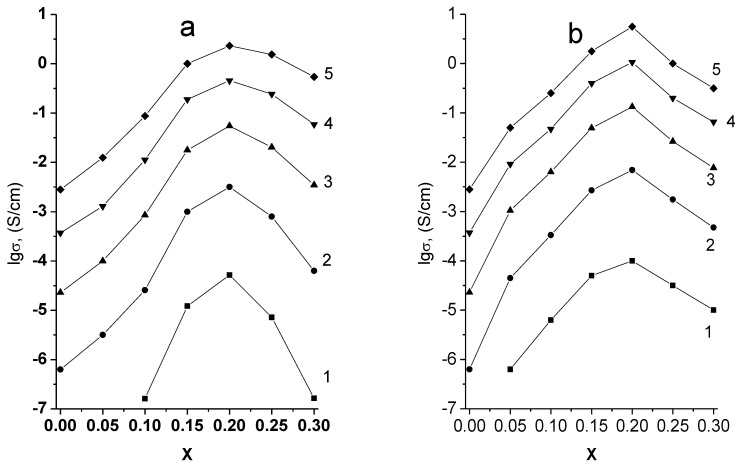
Conductivity isotherms of Li_6-x_Zr_2-x_A_x_O_7_ A = Nb (**a**), Ta (**b**). 1–200; 2–300; 3–400; 4–500; 5–600 °C.

The Arrhenius plots of specific conductivity in both systems studied are linear (Figure 10). The maximum conductivity values in both cases correspond to the samples on the boundaries of single-phase regions (Figure 11, Table 2), i.e., to Li_5.8_Zr_1.8_Nb(Ta)_0.2_O_7_.

The appearance of impurity phases when x ≥ 0.25 is accompanied by a drop of conductivity in both systems. The conductivity of Li_5.8_Zr_1.8_Ta_0.2_O_7_ is 6.92 × 10^−3^ S × cm^−1^ at 300 °C, which is approximately four times higher than the conductivity of Li_5.85_Zr_1.85_Ta_0.15_O_7_ reported in [23] (1.7 × 10^−3^ S × cm^−1^ at the same temperature), which is explained by a higher content of Ta^5+^ and, consequently, a higher concentration of charge carriers, i.e., lithium vacancies. The conductivity of Li_5.85_Zr_1.85_Nb_0.15_O_7_ given in [23], is by an order of magnitude lower than the one of the tantalum-containing sample with the same dopant concentration, which is not quite understandable considering the similarity of the ionic radii, electronegativities, and other characteristics of niobium and tantalum that may affect the conductivity of solid solutions. In the present work, the ionic conductivity of Li_5.8_Zr_1.8_Nb_0.2_O_7_ sample was found to be 3.16 × 10^−3^ S × cm^−1^ at 300 °C, which is close to the conductivity of tantalum-containing sample of the same composition.

**Figure 12:**
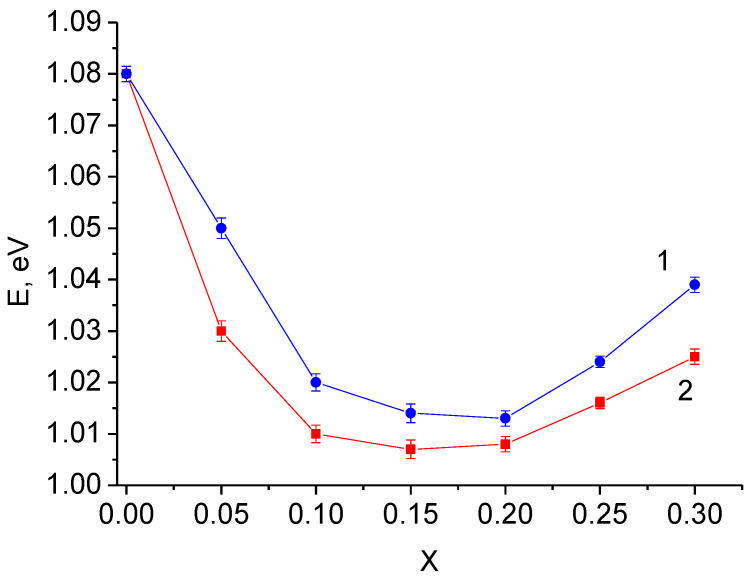
Concentration dependence of activation energy for Li_6-x_Zr_2-x_A_x_O_7_ A = Nb (1), Ta (2).

Solid electrolytes with maximum conductivity in the investigated systems have the lowest activation energy (Figure 12). In the systems with niobium and tantalum these values are similar and equal approximately 1.01 eV, which is close to the values given in literature for vacancy solid solutions based on Li_6_Zr_2_O_7_ [23,25].

Table 3 contains transport properties of the materials investigated in the present paper compared to the properties of other solid electrolytes based on lithium zirconates. One can see that Li_5.8_Zr_1.8_Nb(Ta)_0.2_O_7_ solid solutions have the highest lithium-cation conductivity at 300 °C compared to electrolytes based on Li_2_ZrO_3_ [34], Li_8_ZrO_6_ [20,34], and Li_6_Zr_2_O_7_-based solid solutions that form when lithium is substituted for double-charged cations [22], or when zirconium is substituted for triple-charged cations [24]. It should be noted, however, that the activation energy of Li_6.15_Zr_1.85_Y(In)_0.15_O_4_ interstitial solid solution is much lower compared to vacancy solid solution based on Li_6_Zr_2_O_7_ (Table 3). The authors of [24] assign this to the fact that the coulombic interaction between Li^+^ ions that occupy interstitial positions and the neighbor Li^+^ on normal sites displaces the neighboring normal-site of Li^+^ from the centers of the sites, thus reducing the potential barrier for Li^+^ ions migration. As a result, at 300 °C solid solutions of Li_6.15_Zr_1.85_Y(In)_0.15_O_4_ have a lower conductivity compared to the solid electrolytes studied in the present work, however, at lower temperatures they will apparently have an advantage.

Vacancy solid solutions in Table 3 display the following pattern: solid electrolytes with substitutions in lithium sublattice (Li_1.85_Ca(Zn)_0.075_ZrO_3_, Li_5.6_Mg_0.2_Zr_2_O_7_, Li_7.85_Mg_0.075_ZrO_6_) have a rather lower activation energy compared to the solid electrolytes with substitution of Zr^+^ by Nb^5+^ or Ta^5+^. This can be explained by the influence of the size factor. Activation energy depends on the mobility of the carriers, which, in its turn, depends on the size of crystallographic channels for Li^+^ ions migration. The change of channel dimensions that accompanies heterovalent substitutions depends on the substituted ion and dopant radii. Li^+^ and double charged ions (Mg^2+^, Zn^2+^) have rather close values of ionic radii (0.90, 0.86 и 0.88 Å, respectively [33]). Therefore, the substitution of Li^+^ by Mg^2+^or Zn^2+^ has no significant effect on the mobility of lithium ions. In contrast, Nb^5+^ and Ta^5+^ ions (r = 0.78 Å) are smaller than Zr^4+^ (r = 0.86 Å), so substitution of zirconium by niobium or tantalum leads to a significant decrease in cell volume (Figure 8) and, therefore, in the average radii of channels, which hinders Li^+^ migration and increases the activation energy. However, the maximum dopant solubility in the case of doping Li_6_Zr_2_O_7_ by double charged cations is far less compared to the solubility of the pentavalent ions, so the ionic conductivity of the former is smaller (Table 3).

High lithium-cation conductivity of solid solutions in Li_6-x_Zr_2-x_Nb(Ta)_x_O_7_ systems can be assigned to a relatively open framework structure of the monoclinic Li_6_Zr_2_O_7_ [23] and, compared to other lithium zirconates, a rather high solubility of the dopants, and, consequently, a higher concentration of charge carriers. Nevertheless, compared to the best solid lithium-cation conductors [36], the conductivity of the materials under study is relatively low, therefore, they can be used as solid electrolytes in LIBs only together with additional means of reducing the internal resistance of the battery, e.g., by using solid electrolytes in the form of thin films.

## 4. Conclusions

In the present work, solid lithium-cation electrolytes in Li_6-x_Zr_2-x_A_x_O_7_ (A = Nb, Ta) systems with 0 ≤ x ≤ 0.30 have been synthesized. The electrolytes hold the monoclinic structure of Li_6_Zr_2_O_7_ when x ≤ 0.25. The monoclinic modification of Li_6_Zr_2_O_7_ has been shown to exhibit better transport properties compared to the triclinic one. Temperature (300–600 °C) and concentration dependences of conductivity have been studied for the synthesized materials, their transport properties have been compared with the properties of other lithium zirconate-based solid electrolytes. Solid solutions of Li_5.80_Zr_1.80_Nb(Ta)_0.20_O_7_ composition have a higher lithium-cation conductivity at 300 °C compared to solid electrolytes based on other lithium zirconates and to Li_6_Zr_2_O_7_-based solid solutions yielded by doping Li_6_Zr_2_O_7_ with double- or triple-charged cations. The high conductivity of the solid solutions under study is related to the “open” structure of monoclinic Li_6_Zr_2_O_7_ and high solubility of the doping five-charged cations which provides a high concentration of lithium vacancies. It should be noted, however, that, even though the conductivity of solid electrolytes obtained in this work exceeds the conductivity of Li_2_ZrO_3_- and Li_8_ZrO_6_-based solid solutions, it remains relatively low compared to the best solid lithium-cation conductors, therefore, if the solid solution studied in this paper are to be used as electrolytes in all-solid-state LIBs, the internal resistance of the battery should be reduced by additional means, e.g., by using solid electrolytes in the form of thin films. Additionally, such solid solutions can be used as construction and electrode materials due to their thermodynamic stability in contact with metallic lithium.

## Figures and Tables

**Table 1:** Transport properties of Li_6_Zr_2_O_7_ and its solid solutions.

Solid Electrolyte	σ_Li_^+^, (Scm^−1^) at 300 °C	E*_a_*, (eV)
Li_6_Zr_2_O_7_ [12]	3.0 × 10^−6^	0.98
Li_6_Zr_2_O_7_ [23]	1.2 × 10^−5^	1.25
Li_6_Zr_2_O_7_ [24]	9.4 × 10^−6^	0.84
Li_6_Zr_2_O_7_ [22]	10^−6^	1.07
Li_5.85_Zr_1.85_Nb_0.15_O_7_ [23]	1.8 × 10^−4^	0.99
Li_5.85_Zr_1.85_Ta_0.15_O_7_ [23]	1.7 × 10^−3^	0.95

**Table 2:** Phase composition of Li_6-x_Zr_2-x_A_x_O_7_ (A = Nb, Ta) samples according to XRD.

A	X					
	0.05	0.10	0.15	0.20	0.25	0.30
Nb	ss	ss	ss	ss	ss + Li_29_Zr_9_Nb_3_O_40_	ss + Li_29_Zr_9_Nb_3_O_40_
Ta	ss	ss	ss	ss	ss + ?	ss + ?

ss indicates solid solutions with the monoclinic structure of Li_6_Zr_2_O_7_.

**Table 3:** Transport properties of some solid electrolytes based on lithium zirconates.

Solid Electrolyte	σ_Li_^+^, (Scm^−1^) at 300 °C	E*_a_*, (eV)
Li_5.8_Zr_1.8_Ta_0.2_O_7_, this work	6.92 × 10^−3^	1.01
Li_5.8_Zr_1.8_Nb_0.2_O_7_, this work	3.16 × 10^−3^	1.01
Li_1.85_Ca(Zn)_0.075_ZrO_3_ [34]	~10^−5^	0.85
Li_1.95_Zr_0.95_Nb_0.05_O_3_ [34]	4 × 10^−6^	1.20
Li_2.05_Zr_0.95_Y_0.05_O_3_ [34]	10^−6^	1.24
Li_5.6_Mg_0,2_Zr_2_O_7_ [22]	10^−4^	0.95
Li_7.98_Zr_0.98_V_0.02_O_6_ [35]	3.7 × 10^−5^	1.03
Li_7.85_Mg_0.075_ZrO_6_ [20]	3.5 × 10^−5^	0.85
Li_6.15_Zr_1.85_Y_0.15_O_7_ [24]	8.0 × 10^−4^	0.63
Li_6.15_Zr_1.85_In_0.15_O_7_ [24]	6.4 × 10^−4^	0.69

## Data Availability

The data presented in this study are available on request from the corresponding author.
